# The Acquisition of Complement-Dependent Cytotoxicity by the Type II Anti-CD20 Therapeutic Antibody Obinutuzumab

**DOI:** 10.3390/cancers16010049

**Published:** 2023-12-21

**Authors:** Alicja Kuźniewska, Alan Majeranowski, Sara Henry, Daria Kowalska, Grzegorz Stasiłojć, Aleksandra Urban, Jan M. Zaucha, Marcin Okrój

**Affiliations:** 1 Department of Cell Biology and Immunology, Intercollegiate Faculty of Biotechnology, University of Gdańsk and Medical University of Gdańsk, Dębinki 1 Street, 80-211 Gdańsk, Poland; alicja.kuzniewska@gumed.edu.pl (A.K.); alan.majeranowski@gumed.edu.pl (A.M.); sarahenry48@gmail.com (S.H.); daria.kowalska@gumed.edu.pl (D.K.); gstasilojc@gumed.edu.pl (G.S.); aleksandra.urban@gumed.edu.pl (A.U.); 2 Department of Hematology and Transplantology, Medical University of Gdańsk, Smoluchowskiego 17 Street, 80-214 Gdańsk, Poland; jan.zaucha@gumed.edu.pl

**Keywords:** complement system, rituximab, obinutuzumab

## Abstract

**Simple Summary:**

Therapeutic anti-CD20 monoclonal antibodies (mAbs) are divided into two types based on their dominant effector mechanism. In contrast to type I specimens, obinutuzumab, a representative of type II mAbs, poorly activates the complement system. Recent studies explained that the structure of the antigen–antibody complex characteristic for type II antibodies precludes oligomer formation, which otherwise supports an efficient complement activation by human mAbs. Herein, we provide evidence that obinutuzumab’s ability to activate complement can be rescued at later stages of the cascade, as observed in the presence of hyperactive complement convertase components. Such modulation, which enforces additional effector mechanisms, may be an alternative way of improving a killing repertoire of already existing drugs rather than designing their novel versions.

**Abstract:**

Rituximab, a prototypic anti-CD20 mAb, and the third-generation anti-CD20 mAb obinutuzumab differ in their ability to activate the complement system. According to recent studies, this contrast stems from the architecture of the antigen–antibody complex formed by these two mAbs that facilitates (rituximab) or disables (obinutuzumab) further oligomerization, leading to engagement of the initial classical complement pathway component C1q. We examined whether a gain-of-function C2 variant that acts downstream of C1q and enforces the formation of complement convertase resistant to physiological decay can impact complement activation by obinutuzumab. Co-application of the C2 variant with obinutuzumab and human serum resulted in complement-dependent cytotoxicity equal to or higher than attainable for rituximab. This effect was observed either in serum or hirudin-anticoagulated whole blood. Long-term (24 h) overall cytotoxicity of obinutuzumab was improved in target cells of moderate sensitivity to complement but diminished in cells of low sensitivity. Our results demonstrate that the ability of complement activation of a given antibody is not ultimately determined at the stage of initial interactions with its target antigen but is modulable at later stages of the cascade and that the benefit of the acquisition of this new effector mechanism by obinutuzumab depends on the target cell characteristics.

## 1. Introduction

Since the approval of rituximab by the Food and Drug Administration in 1997, anti-CD20 monoclonal antibodies (mAbs) have been widely used in clinics [1,2]. Their original indication included the treatment of B cell malignancies such as non-Hodgkin’s lymphomas and chronic lymphocytic leukemia (CLL), later extended to autoimmune conditions like rheumatoid arthritis, pemphigus vulgaris, microscopic polyangiitis, and granulomatosis with polyangiitis [3]. Targeting the CD20 molecule results in the elimination of normal and malignant B cells, which can be mediated either through direct induction of cell death or host immune system-supported processes. The prevalent effector mechanism grounds the anti-CD20 mAbs’ classification into two types: type I (e.g., rituximab, ofatumumab), characterized by superior complement activation and poor direct induction of cell death, and type II (e.g., obinutuzumab) with the opposite characteristics [4]. During the past twenty years, parameters like the proximity of the target epitope to the cell membrane, the segregation of mAbs into the lipid rafts, and the off-rate of mAbs were believed to influence the effector mechanism of the antibody [5]. However, in 2020, the publication in Science by Kumar et al. shed a new light on the differences between type I and type II anti-CD20 mAbs exemplified by rituximab and obinutuzumab, respectively. The binding of a type I mAb to its target antigen occurs in a way that facilitates the recruitment of additional mAb particles into the complex and eventually leads to the formation of oligomers [6]. An oligomeric (or ideally, hexameric) structure fits the first component of the classical complement pathway, C1q. For instance, binding the first particle of a type II mAb to its target precludes oligomerization and results in weak complement engagement. This hypothesis was further confirmed by introducing a mutation into the constant part of the heavy chain of human IgG antibodies that results in enforced oligomerization of mAbs. Unlike their original version, such modified antibodies showed substantial complement-dependent cytotoxicity (CDC) [7]. The abovementioned results revisited the theories on parameters that drive type I or type II characteristics and depicted the structure of the antigen–antibody complex as a decisive factor. Herein, we elucidate whether the interference downstream from the antigen–antibody complex may turn a type II mAb into a strong complement activator. Based on the homology of the alternative and classical complement pathway paralogs, we translated known gain-of-function (GoF) mutations in the gene coding for the alternative pathway component factor B into the classical complement pathway component C2 [8]. Supplementation of a serum with such an in silico-designed GoF variant of C2 resulted in markedly increased complement-dependent cytotoxicity (CDC) initiated by type I anti-CD20 antibodies rituximab and ofatumumab due to the formation of hyperactive classical complement convertases [8]. Now, the emerging question was whether the type II anti-CD20 mAb obinutuzumab acting in concert with the GoF C2 variant can exert substantial CDC, despite the suboptimal structure of the antigen–antibody complex.

## 2. Materials and Methods

### 2.1. Cell Lines and Fresh Cultures of Chronic Lymphocytic Leukemia (CLL)

Previously, we characterized twelve human leukemia and lymphoma cell lines regarding their susceptibility to CDC initiated by type I anti-CD20 mAbs and found that such vulnerability was determined by the ratio of the target molecule CD20 to the membrane-bound complement inhibitors, mainly CD55 and CD59 [9]. Three of these cell lines of different sensitivity to CDC: Namalwa (low sensitivity), Raji (moderate sensitivity), and Ramos (high sensitivity) were obtained from ATCC and cultured in RPMI1640 medium with L-glutamine supplemented with 10% fetal bovine serum (both from ATCC) in a humidified 5% CO_2_ atmosphere. Fresh CLL cells were isolated from the treatment-naïve patients admitted to the Department of Hematology and Transplantology of the Medical University of Gdańsk and previously diagnosed with chronic lymphocytic leukemia based on specific markers and cell count (we collected material from patients with a total clonal lymphocytosis >10^5^/mm^3^, which was 20 times higher than the minimal count for CLL diagnosis). Blood was drawn into a sodium citrate tube (Vacutainer) then diluted twice with a sterile PBS buffer, loaded onto a lymphoprep gradient, and centrifuged for 15 min at 700 rpm. Buffy coats were collected, washed twice with PBS, and suspended in a 1:1 mixture of RPMI1640 and DMEM medium (Hycult) supplemented with 20% fetal bovine serum (PanBiotech, Aidenbach, Germany). The next day, cells were analyzed by flow cytometry and used for experiments when the count of CD20-positive events was higher than 90%.

The patients and healthy blood donors signed a written informed consent to participate in the study, which was conducted in accordance with the Declaration of Helsinki and with approval from the Local Bioethical Committee at the Medical University of Gdańsk (approval number: NKBBN/500/2016).

### 2.2. GoF Variant of Complement C2 Protein

The variant of complement C2 protein containing the amino acid substitutions C261A, Q263G, Y347A, and T442Q was expressed as a C-terminal His-tagged protein in the eukaryotic HEK293Freestyle system and purified by nickel-affinity chromatography (Ni-NTA fast flow column, GE Healthcare) as described in [8]. Purified protein was aliquoted and kept at −80 °C until the experiments.

### 2.3. CDC and Classical Complement Convertase Assays

CDC and convertase assays were performed as described elsewhere [8,10]. Briefly, for the CDC assay, cells were loaded with calcein AM for 30 min, then washed and incubated with anti-CD20 antibodies (50 μg/mL) and normal human serum at 1.25% (Ramos), 10% (Raji), or 20% concentration (Namalwa and CLL isolates) diluted in PBS with 1 mM Ca^2+^/Mg^2+^, respectively. A different serum concentration for each cell line (established based on our results published previously in [9]) aimed to provide an optimal assay window; otherwise, the effect of supplementation with the GoF C2 variant would be missed in cell lines highly sensitive to CDC. After 30 min, cells were centrifuged, and the supernatant was transferred to a flat-bottom microplate. The percentage of lysis was assessed by the measurement of fluorescence at 490/520 nm and compared to the readout from the cells incubated in 30% DMSO (considered as full lysis). For the convertase assay, calcein-AM-loaded cells were incubated with anti-CD20 antibodies diluted in 10% C3-depleted serum (for C3 classical convertase assay) or C5-depleted serum (for C5 classical convertase assay). After the indicated time period (from 1 to 30 min), the cells were washed and incubated with guinea pig serum diluted in 40 Mm EDTA-containing veronal buffer for 30 min. The fluorescence of the supernatant was measured as for the CDC assay.

### 2.4. XTT Assay

Cells (10^5^/well) were washed and suspended in RPMI medium supplemented with 10% fetal bovine serum (control cells) in a flat-bottom, 96-well plate. The cells in the other wells were additionally supplemented with normal human serum (1.25% for Ramos, 5% for Raji, and 20% for Namalwa cells) and optionally with the therapeutic antibody rituximab or obinutuzumab at 50 μg/mL and respective concentrations of the GoF C2 variant that corresponded to the physiological level of C2 in normal human serum (25 μg/mL). Cells were cultured for 24 h, then an XTT and PMS (phenazine methosulfate) (both from Thermo Fisher Scientific, Waltham, MA, USA) solution mix (50:1 *v*/*v*) was added, according to the manufacturer’s protocol. Following 90 min of incubation, the supernatant was transferred to another flat-bottom microplate, and the absorbance was measured at 450 nm with a 690 nm correction.

### 2.5. Whole Blood CDC Assay

Samples of 4 mL of blood were drawn from healthy volunteers and immediately mixed with hirudin/lepirudin (Sigma Aldrich, Saint Louis, MO, USA), currently the only available anticoagulant that does not affect complement activity [11], to the final concentration of 30 U/mL. The blood was kept on ice until the experiment, which from this point principally followed the protocol for CDC assay.

## 3. Results

### 3.1. CDC Assays

**Figure 1:**
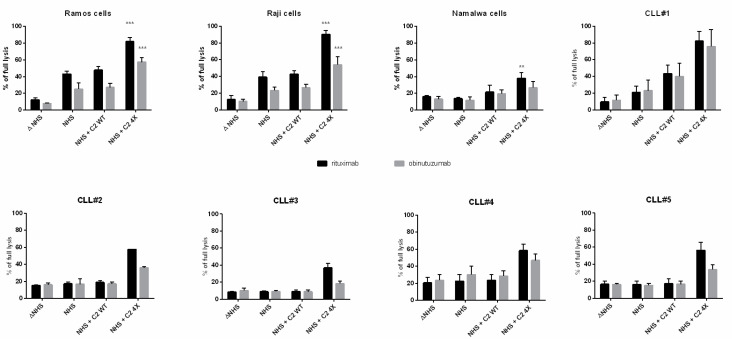
CDC assay on human CD20+ cells of different sensitivity to anti-CD20 mAbs and CLL cells freshly isolated from patients. The CDC assay was performed in the presence of normal human serum (NHS) supplemented with rituximab or obinituzumab +/−C2 wild type (WT) or the GoF variant (4X). The readout obtained for the cells incubated in heat-inactivated normal human serum (Δ NHS) was considered as a negative control, i.e., a background lysis that was not connected to complement activity. Full lysis was considered as a readout obtained from cells lysed with 30% DMSO. Data for cell lines are the averages from three independent experiments (*n* = 3), and the symbols ** and *** depict statistically significant differences between cells supplemented with 4× and the WT variant of C2 at *p* < 0.01 or *p* < 0.001, respectively, according to Sidak’s multiple comparison test. Error bars show standard deviation (SD). Data from freshly isolated CLL cells present the average from three technical repetitions.

Previously, we characterized several human CD20+ cell lines according to their sensitivity to type I anti-CD20 antibodies [9]. Three of these cell lines of high (Ramos), moderate (Raji), and low (Namalwa) sensitivity were selected for the current experiments. In each of the cell lines tested, the addition of hyperactive C2 to obinutuzumab resulted in an increase of complement-mediated lysis that outperformed the readout obtained for cells sensitized with the type I anti-CD20 mAb rituximab (Figure 1). Importantly, no effect was observed when wild-type C2 was used instead of the GoF variant. In the same experimental setup, the GoF variant added to the serum without anti-CD20 mAbs caused no significant cytotoxicity (Supplementary Figure S1). The same experiment was repeated on fresh cultures of CLL cells obtained from the patients. CLLs are considered relatively resistant to the CDC initiated by type I anti-CD20 mAbs and represent a model closely related to challenges met during clinical practice. We examined CLL cells from five donors, and in all cases, we observed the same phenomenon as in the experiments with cell lines, i.e., enforcement of CDC initiated by obinutuzumab supplemented with the GoF C2 variant, which yielded higher lysis than obtained with rituximab with no C2 supplementation.

### 3.2. Convertase Assays

**Figure 2:**
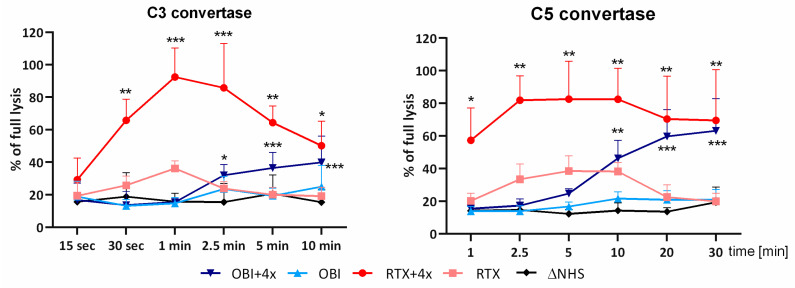
The activity of classical complement convertases formed on the platforms of rituximab and obinutuzumab. Classical C3 (left graph) and C5 (right graph) convertases were formed on the surface of Ramos cells incubated with 10% normal human serum (NHS) supplemented with obinutuzumab (OBI) or rituximab (RTX) in two conditions: with and without addition of the hyperactive C2 variant (4×). Data are averages from three independent experiments (*n* = 3), and the symbols *, **, and *** depict statistically significant differences between cells treated with the given antibody supplemented with 4× and the WT variant of C2 at *p* < 0.05, *p* < 0.01, or *p* < 0.001, respectively, according to Sidak’s multiple comparison test. Error bars show standard deviation (SD).

Next, we wanted to confirm that the GoF C2 variant that supports the efficient CDC activity of obinutuzumab acts at the level of complement convertases. To prove that, we performed a convertase activity assay, in which the function of the enzymatic complex was measured against time (Figure 2). Under normal circumstances, the convertase complex activity rises soon after cascade stimulation, reaches the point of maximal activity (so-called T_max_), and decreases to the background level [12]. Compounds that provide convertase hyperactivity can either increase the T_max_ value or keep the complex active for a prolonged period. This was observed when the GoF C2 variant was added to rituximab (Figure 2). Importantly, the T_max_ of the convertase complex formed upon stimulation with obinutuzumab and the hyperactive C2 variant was achieved at a longer time point compared to analogic conditions with rituximab, but eventually, the amplitude of the C5 convertase activity reached the level attainable for a type I anti-CD20 mAb.

### 3.3. Whole Blood CDC Asssys

**Figure 3:**
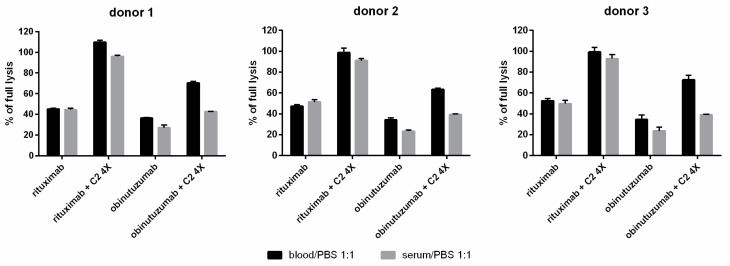
Whole blood CDC assay. Blood and serum from three individuals were used in the CDC assay performed in Raji cells. The principles and technical details of the assay are the same as those presented in Figure 1. Presented data show the averages from two technical repetitions. Error bars show standard deviation (SD).

The results of in vivo experiments in mouse mAb-sensitized lymphoma cells showed that complement activation and Fc-receptor-mediated effector mechanisms such as ADCC are competitive with each other [13]. In vitro study suggests that at the level of a single target cell, fixation of early complement components constitutes a steric hindrance that disables the adhesion of NK cells to their target via CD16/FcγRIII [14]. However, in practice, these two mechanisms may act in concert when the population of tumor cells is heterogeneous in terms of the expression of target antigens or when the availability of effector cells is not insufficient regarding the tumor burden. Van Meerten and coworkers showed that the threshold of CD20 expression necessary for CDC activation is substantially lower than the threshold for effective engagement of NK cells [15]. Therefore, we attempted to find out whether the enhancement of CDC initiated by obinutuzumab in the presence of a hyperactive C2 variant will persist in a whole blood environment when FcR-bearing cells are present. For this purpose, blood coagulation was performed with hirudin, which does not affect complement activity, unlike other commonly used anticoagulants [11]. We examined blood collected from three individuals, and, on the same occasion, we separately examined sera collected from the same people (Figure 3). The experiment performed on Raji cells revealed that upon addition of the GoF C2 variant to whole blood, the enhancement of CDC initiated by obinutuzumab was observed. Surprisingly, such an enhancement outperformed the analogous increase observed in serum, whereas the CDC ratio in rituximab-treated cells was similar in whole blood and serum. This result suggests that in the context of short-term cytotoxicity, obinutuzumab may benefit from complement as its additional effector mechanism in the physiological environment of whole blood.

### 3.4. Long-Term Cytotoxicity

**Figure 4:**
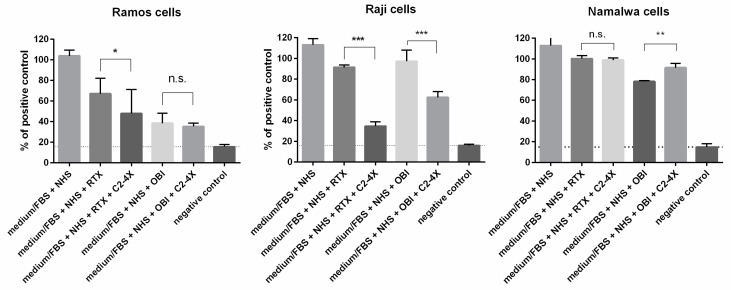
XTT assay performed for 24 h in complete culture medium. The principles of the XTT assay were to enable the proliferation of cells that resisted CDC and, at the same time, to expose them to the direct cytotoxic effect of anti-CD20 mAbs. The background (negative control, dashed lines) level was considered as the XTT conversion in medium without cells, and the positive (100%) control was set as the readout obtained for cells kept in complete medium without human serum. Data are averages from at least three experiments (*n* ≥ 3), and the symbols *, **, and *** depict statistically significant differences between cells treated with the given antibody supplemented with 4× and the WT variant of C2 at *p* < 0.05, *p* < 0.01, or *p* < 0.001, respectively, according to Sidak’s multiple comparison test. Error bars show standard deviation (SD).

The hallmark of type II anti-CD20 antibodies is the execution of target cell death by direct mechanisms [4]. Therefore, our next question was whether the enforced complement activation by the hyperactive C2 variant interferes with the induction of cell death by rituximab or ofatumumab. For this purpose, we performed a 24 h experiment (unlike the 30 min CDC assay) in a complete cell culture medium to enable a long-term direct effect and to assess both the net result of killing due to CDC (taking place during the first minutes of the experiment) and the proliferation of complement-resistant cells. Out of three cell lines tested, those of high sensitivity to CDC were killed more efficiently by the combined action of rituximab and the GoF C2 variant, and no significant changes were observed when obinutuzumab was applied (Figure 4). The cells of moderate sensitivity to CDC showed the additive effect of enforced complement activation and the direct effects of both mAbs. Interestingly, highly complement-resistant Namalwa cells were killed less efficiently when obinutuzumab was combined with the hyperactive C2 variant.

## 4. Discussion

Complement activation by antibodies is the multistep process that consists of the binding of pattern-recognition molecules such as C1q, conformational changes within the C1 complex that release proteolytic activity of C1r and C1s molecules, the cleavage of C4 and C2, the formation of convertases that augment the pathway by producing C3b and C5b, and finally the assembly of the membrane attack complex [16]. Convertases are labile complexes that upon formation quickly reach a peak of their activity and then undergo physiological decay, either spontaneous or fueled by membrane-bound and soluble complement inhibitors. Importantly, half of the proteins identified as the complement system components act as inhibitors, and the majority of them control complement convertases, thus indicating that this is a pivotal step in the entire cascade. Indeed, dysregulation leading to loss of control of the convertase activity results in complement-driven auto-degenerative diseases such as C3 glomerulopathy (C3G), atypical hemolytic uremic syndrome (aHUS), age-related macular degeneration (AMD), or paroxysmal nocturnal hemoglobinuria (PNH) [17]. Dysregulation may involve the appearance of loss-of-function genetic variants in complement inhibitors and gain-of-function (GoF) variants in convertase components, which render these complexes more stable and/or insensitive to regulation. Previously, based on the known GoF genetic variants of complement factor B (a component of the alternative complement pathway) and its high amino acid sequence homology to its classical complement pathway analog [18], we designed a hyperactive C2 variant with three substitutions: p. Q263G, Y347A, and T442Q. When added to human serum and reacted with lymphoma cells opsonized with the type I anti-CD20 mAbs rituximab and ofatumumab, the recombinant triple C2 mutant formed convertases of higher activity and extended stability compared to those built from the wild-type C2 protein [8]. The overall effect of the hyperactive C2 addition was elevated deposition of C3 on target cells and markedly increased CDC initiated by the type I anti-CD20 mAbs rituximab or ofatumumab. Herein, we modified our GoF C2 construct by the addition of another amino acid substitution, C261A, claimed to improve the intrinsic stability of classical convertase [19], and used such a quadruple C2 mutant to check whether the complement cascade enforcement at the stage downstream of the antigen–antibody complex can effectively increase CDC on the platform of the type II anti-CD20 mAb obinutuzumab. Obinutuzumab is a humanized, glycoengineered monoclonal antibody, which, due to the lack of fucose sugar residue in its Fc part and to modifications in the elbow-hinge region, shows, on the one hand, a superior affinity to the FcγRIII receptor and, on the other hand, a diminished rate of FcγRIIb-mediated internalization of mAb–CD20 from the cell membrane [20]. These features promote FcR-mediated effectors and direct effects on target cells and concurrently diminish the potential of complement activation. Conversely, rituximab and ofatumumab recruit substantial amounts of C1q to the surface of target cells causing rapid massive deposition of C3b and subsequent cell death [21]. Many molecular attributes like the proximity of the target epitope to the cell membrane, the density of antigens, the off-rate of the antibody, and the ability to cluster into the lipid rafts were all considered important in predisposition toward a particular type’s characteristics [22]. However, Kumar and coworkers revealed that the structure of the antigen–antibody complex is pivotal for efficient complement activation and explains differences between rituximab and obinutuzumab regarding the preferred effector mechanism [6]. Type I antibodies bind in a way that establishes 1:2 or 2:1 antibody–antigen “seeding” complexes that enable further oligomerization, whereas type II specimens establish 1:1 or 2:2 “terminal” complexes that preclude additional expansion. Our results suggest that despite an antigen–antibody complex architecture suboptimal for complement activation, type II anti-CD20 mAbs can bind the C1 complex and activate further steps of the complement cascade, leading to the formation of a low number of convertase complexes. We postulate that these complexes are quickly turned inactive by numerous complement inhibitors before producing a substantial amount of C3b. However, the formation of convertases from the C2 component resistant to inactivation overwhelms the inhibitors and enables the complement cascade to eventually trigger CDC. This scenario is justified when one looks at the delay in reaching maximal activity of C3 and C5 convertases built on the platform of obinutuzumab in the presence of the quadruple C2 mutant (Figure 2).

By showing the modulation of obinutuzumab towards efficient CDC that takes place downstream of the antigen–antibody complex, our results can establish GoF C2 variants as molecular tools for studying the reasonableness of entailing all immune effector functions to a single antibody. Type I and II anti-CD20 mAbs have different limitations. Unproductive complement consumption, fueled by inhibitors overexpressed on the surface of tumor cells and/or hijacked by tumor cells from serum, limits the efficacy of type I specimens [23,24]. The activity of type II mAbs depends on the functionality of intracellular signaling cascades and the number of available effector cells (NK cells, macrophages), which can be inefficient at a high tumor burden, unlike the complement components that are ubiquitous in blood and penetrate easily to extravascular locations. There are efforts to produce type III mAbs that share type I and type II characteristics, and a few such compounds exist in the preclinical development stage [25,26,27,28]. However, modification of existing, clinically approved type I or type II anti-CD20 mAbs to achieve type III features sounds challenging since there are no structural studies on type III mAb complexes with the CD20 antigen yet, which could be a hint for drug designers. Only a small portion of the CD20 molecule constitutes the extracellular face of the CD20 antigen [29]. Dozens of anti-CD20 monoclonal antibodies reported in the literature have been raised, and despite binding to the overlapping epitopes, they often differ in type I/type II properties [30]. Therefore, even a slight modification of antibody structure may be critical for its functionality. Supplementation of serum with GoF C2 variants does not interfere with the antigen–antibody complex but enforces the CDC activity of antibodies that normally do not activate complement, which enables a clear-cut comparison of original vs. acquired properties of therapeutic mAbs in, e.g., an in vivo model. A rationale for extended experiments including animal studies is brought by our results presenting the GoF C2 efficacy in a whole blood environment (Figure 3) but also by the results of short- and long-term cytotoxicity observed in cell lines of low- and high susceptibility to complement (Figure 1 vs. Figure 4), suggesting that the benefits from CDC acquired by type II mAbs are model-dependent.

## 5. Conclusions

Based on the results of CDC and convertase activity assays, we conclude that obinutuzumab recruits complement components less efficiently than rituximab, but supported with compounds that form more stable classical convertases, it executes a delayed but enhanced complement-mediated lysis. Nonetheless, the most important conclusion from this study is that the structure of the antigen–antibody complex is important but not the ultimate issue that determines efficient complement activation, as the augmentation of the complement cascade downstream of antibody binding may enforce efficient CDC from a type II anti-CD20 mAb. The GoF C2 variant’s application opens technical possibilities of adding complement activation as an extra effector mechanism to type II mAbs. Notably, our in vitro results suggest that such modulation may be beneficial or contra-productive for the overall long-term cytocidal effect of obinutuzumab, depending on the basal susceptibility of the tumor cell to complement-mediated lysis. Assuming that the characteristic of an appropriate target can be defined and that personalized medicine enables a detailed selection of patients, broadening the killing mode repertoire of already established immunotherapeutics emerges as an attractive avenue of drug development. However, the advantages and disadvantages of such type II mAb modulation must be verified in separate in vivo studies.

## Data Availability

Raw data are available upon request from the corresponding author.

## Supplementary Materials

The following supporting information can be downloaded at https://www.mdpi.com/article/10.3390/cancers16010049/s1: Figure S1: Impact of C2 variant on short-term cell cytotoxicity.
