# Supplementary material for: The Acquisition of Complement-Dependent Cytotoxicity by the Type II Anti-CD20 Therapeutic Antibody Obinutuzumab
Source: Cancers (Basel). 2023 Dec 21;16(1):49. doi: 10.3390/cancers16010049 (PMC10778491; doi:10.3390/cancers16010049)
Supplement: Supplementary file 1 [file cancers-16-00049-s001.zip › Supplemetary Figure S1.pdf]

Supplemetary Figure S1. Impact of C2 variant on short-term cell cytotoxicity.

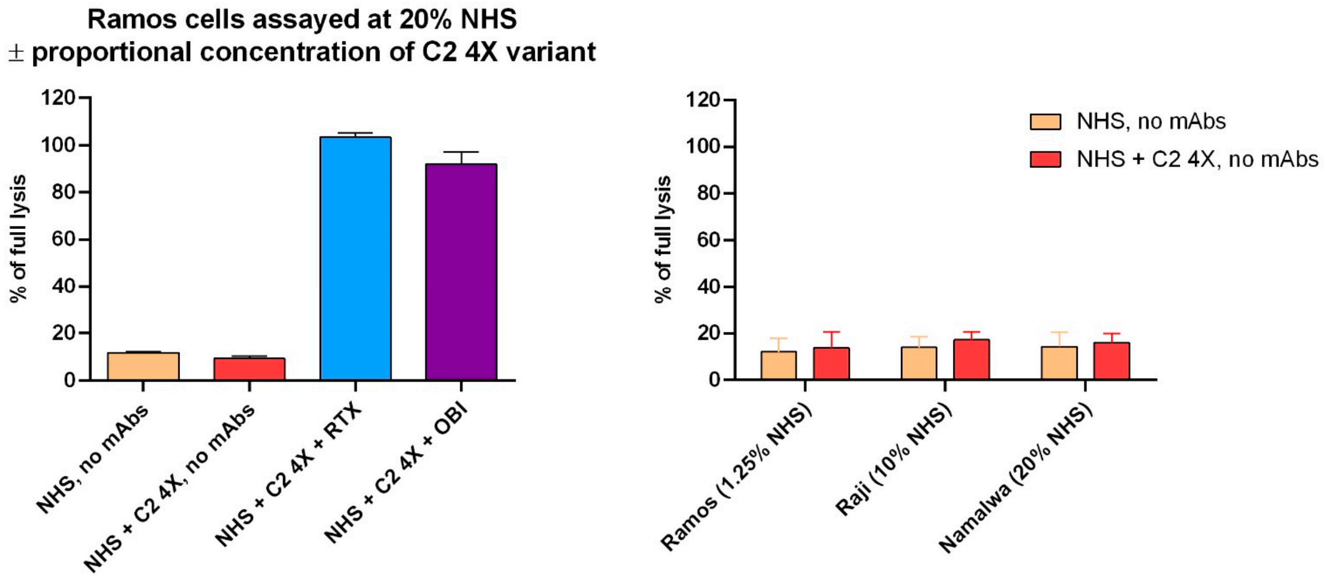

Additional negative controls for the CDC assays.

Left graph: Calcein release assay was performed upon the same parameters (cell number, incubation time, calcein-AM load) as in Fig.1 and Fig.3. Ramos cells were incubated at the maximal serum concentration used in Fig. 1 (20% NHS, which was used to perform CDC in Namalwa cell line, i.e. 8 times higher than normally used for Ramos cells). The tested conditions were NHS alone, NHS supplemented with gain-of-function variant of C2 (C2 4X) proportionally as for 20% serum (i.e. 8 times more than normally used for Ramos cells) but without anti-CD20 mAbs added, NHS supplemented with C2 4X and standard concentrations of rituximab or obinutuzumab (50 mg/ml). Right graph: Assays testing the cytotoxic potential of the C2 4X variant diluted in NHS without anti-CD20 mAbs vs. NHS alone were performed on Ramos, Raji, and Namalwa cells at 1.25%, 10%, or 20% NHS proportionally supplemented with hyperactive C2 protein (the same conditions as in Fig. 1 and 3).
